# Rivastigmine as a Symptomatic Treatment for Apathy in Parkinson's Dementia Complex: New Aspects for This Riddle

**DOI:** 10.1155/2017/6219851

**Published:** 2017-03-19

**Authors:** Rita Moretti, Paola Caruso, Matteo Dal Ben

**Affiliations:** ^1^Clinica Neurologica, Dipartimento Universitario Clinico di Scienze Mediche, Chirurgiche e della Salute, Università degli Studi di Trieste, Trieste, Italy; ^2^FIF Science Park, University of Trieste, Trieste, Italy; ^3^Dipartimento Universitario Clinico di Scienze Mediche, Chirurgiche e della Salute, Università degli Studi di Trieste, Trieste, Italy

## Abstract

Over 90% of PDD patients show at least one neuropsychiatric symptom (NPS); in the 60–70% two or more NPS are present. Their incidence is important in terms of prognosis and severity of pathology. However, among all NPS, apathy is often the most disturbing, associated with greater caregiver's burden. Similar to other NPS, apathy may be due to a dysfunction of the nigrostriatal pathway, even though, not all the PD patients become apathetic, indicating that apathy should not entirely be considered a dopamine-dependent syndrome, and in fact it might also be related to acetylcholine defects. Apathy has been treated in many ways, without sure benefits; among these, Rivastigmine may present benefic properties. We present a series of 48 patients, suffering from PDD, treated with Rivastigmine, and followed-up for one year; they have been devotedly studied for apathy, even though all the other NPS disorders have been registered. Rivastigmine did not have a prolonged benefic effect on apathy, in our work, on the contrary of what had been observed in the literature, probably due to the longer follow-up of our patients.

## 1. Introduction

Behavior and cognitive symptoms are common in Parkinson's Disease (PD) and in Parkinson's Disease Dementia (PDD) [1–6]. As well pointed out by a recent study [7], 90% of PDD patients showed at least one neuropsychiatric symptom (NPS) and a percentage up to 60–70% two or more NPS [8]; NPS are important predictive factors for prognosis, institutionalization, and overall mortality [9–13]. There is no clinical consensus on how to treat NPS; antipsychotics drugs are widely employed, but they should be used only for small amount of time, and they are recommended to treat hallucinations, delusions, and aggressiveness. Major warning has been given by FDA to atypical neuroleptics [8] and the American Geriatric Society (AGS) Beers consensus criteria for safe medication use in the elderly [14] recommend avoiding antipsychotics to treat NPS of dementia due to the increased mortality and CVAE risk [14, 15]. Cholinesterase and butirrylcholinesterase inhibitors and NMDA antagonists have been used to treat primary cognitive disturbance in PDD [16], but there are some data, which give reason for their benefits also in the management of NPS [17–21].

However, among all the NPS, one of the most intriguing (for the complicate pathophysiological mechanism underlying it) [22] and one of the most disturbing (for caregivers and for patients) is apathy. The presence of apathy has been associated with greater cognitive impairment [[23–27]; see data in [22]], and its prevalence in PDD varies between 16.5% and 51%, depending upon the instrument for assessment and on the samples examined [28–30]. It has been hypothesized that dysfunction of the nigrostriatal pathway might be involved in the pathophysiology of apathy in PD, [29], confirmed by functional connectivity study which documented a conspicuous impairment of striatal and ventrolateral prefrontal regions connections [31]. Data are not univocal, since two other studies [32, 33] did not find out any structural differences when comparing apathetic to nonapathetic PD patients, after applying appropriate correction for multiple comparisons [34].

To be precise, the extension of brain networks involved in apathy in PDD is enormous, and many other neuroimaging and functional studies indicate different brain areas involvement, not only nigrostriatal pathways [data and literature in [22]] documented it. Reijnders et al. [25] found an association between higher apathy and lower gray matter density in the bilateral inferior frontal gyrus and precentral gyrus, in the bilateral inferior parietal gyrus, and right precuneus, confirmed by Skidmore et al. [35], who showed that the severity of apathy was best predicted by a greater sufferance of the right middle orbitofrontal cortex and bilateral subgenual cingulate cortex, of the left supplementary motor cortex, and of the left inferior parietal lobule and left fusiform gyrus [35]. (FDG) PET-studies specifically found a positive correlation of apathy and cerebral metabolism during rest in the right middle frontal gyrus, right inferior frontal gyrus, left anterior insula [26], bilateral orbitofrontal lobes and bilateral anterior cingulate [32], and left posterior cingulate cortex [26]. Much more interesting is that not all the PD patients become apathetic, indicating that apathy should not entirely be considered a dopamine-dependent syndrome in PD, and is in fact present even in not-purely dopaminergic alterations [11, 36, 37]. As strongly pointed out by Kos et al., [34] an inverse correlation between catecholaminergic binding potential, indicative of a specific combined loss of dopamine and noradrenaline innervation, and apathy was found in the bilateral ventral striatum in an exploratory resting-state analysis in PD [38]. Some studies tried to involve acetylcholine in driving motivation and its lack related to apathy [39].

Apathy has been treated in many different ways [17, 40–42]. Some recently published studies [5, 6] suggested some benefic properties of Rivastigmine on this NPS symptom too, in complete accordance with the results obtained by Devos et al. [17].

We present a series of patients, suffering from PDD, treated with Rivastigmine, and devotedly studied for apathy, within a complex sequence of NPS disorders; we did not have the same successful results showed by the previous studies; we discuss the results, trying to give pathological explanation to a different possible mechanism outstanding apathy in PDD.

## 2. Materials and Methods

50 patients diagnosed with PDD, from 1 December 2010 up to 31 December 2013, referring to the Neurological Unit Research of Trieste, were enrolled.

The diagnosis was based on UK PD Society Brain Bank clinical diagnostic criteria and clinical diagnostic criteria for probable PDD [43–45]. Data from a physical and neurological examinations, laboratory tests, and brain magnetic resonance imaging (or CT for 6 patients, claustrophobic and therefore not possible for them to attend MRI) were obtained. Patients with a history of stroke or brain hemorrhage or other psychiatric disorders, atypical PD, or secondary Parkinsonism have not been enrolled for this study.

The patients must completely fulfill the criteria for probable PDD, as presented by Goetz et al. [45]: core features must be present, as well as a typical profile of impaired attention, fluctuation of executive functions, an impairment in visuospatial functions, or impaired free recall; it should be associated with at least one behavioral symptom (such as apathy, depression, and anxiety). Patients must fulfill the diagnostic rating sheet for probable PDD, with a history of PD, with a PD disease developed before dementia, with MMSE' scores less than 26, with an impairment in ADLs, with impaired cognition for the 4 items (sevens backwards; lexical fluency; MMSE pentagons; 3-word recall), with absence of major depression; absence of delirium; and absence of other neuropsychiatric diseases [46].

All patients were diagnosed for the first time as PDD, upon enrollment in this study.

All patients were on antiparkinsonian medications.

The equivalent daily dose of levodopa was calculated as the international standard converting measure [47] as follows: dose of levodopa plus dose of dopamine-agonists multiplied by equivalents (= 1 × levodopa dose + 0.75 × controlled release dose + 0.33 × entacapone + 20 × ropinirole dose + 100 × pramipexole + 10 × selegiline + 1 × amantadine) [47]. Each caregiver's patient gave informed consent for participation before entry. All procedures complied with ethical standards for human investigations and the principles of the Declaration of Helsinki.

### 2.1. Outcome Measures

This was a prospective, longitudinal, open-label, observational, single center, 12-month clinical trial on the effect of Rivastigmine for improving BPSD, with particular reference to apathy. Baseline second level testing data were obtained before starting Rivastigmine, which has been titrated to all patients for eight weeks. All subjects were administered a maintenance dose of Rivastigmine for 12 months. The main outcomes of the study were as follows:

1. Global performance was assessed using the Montreal Cognitive Assessment (MoCA) [48–50]; the test comprises 6 parts, which have been administered in extenso (memory recall-5 scores; visuospatial construction 4 scores; executive functions 4 scores; attention and working memory 6 scores; language 5 scores; orientation: 6 scores). We considered the results as a whole, and not by subscores. The most significant parts, however, are attention, executive function, and visuospatial construction.
2. Executive functions, attention, judgment, and analogical reasoning were assessed by Frontal Assessment Battery (FAB) [51].
3. Apathy was assessed by the Clinician/Researcher Rated Version of the Apathy Evaluation Scale (AES-C) and the parallel Self-Report Version of the same instrument (AES-S) [52]; total score ranges from 18 to 72 points (higher score indicates more severe apathy). The cutoff score ≥ 37 was used to divide apathetic from nonapathetic patients. In the present study, we considered to be apathetic a patient who showed a total AES-S score ≥37.
4. Global behavioral symptoms were assessed by the Neuropsychiatric Inventory, NPI [53]; symptom frequency was rated on a scale of 1 to 4 (1 = less than once a week; 2 = once a week; 3 = several times a week; 4 = everyday), and severity was rated on a scale of 1 to 3 (1 = mild; 2 = moderate; 3 = severe). A composite score ranging from 1 to 12, defined as the product of frequency and severity, was calculated. The important aspect of caregiver distress was also recorded and scored for each neuropsychiatric symptom complex (as the study by Oh et al., 2015-A) [6]. The caregiver was asked to rate their own emotional or psychological distress caused by each symptom on a scale of 0 to 5 (0 = no distress; 1 = minimal; 2 = mild; 3 = moderate; 4 = moderately severe; 5 = very severe). A total caregiver distress score was obtained by summing the individual scores on the 12 items (as the study by Oh et al., 2015-A [6]).

### 2.2. Statistical Analyses

Statistical analyses were performed using the Statistical Package for the Social Sciences (SPSS, version 16.0). Within group changes from baseline to 12 months were tested using the Wilcoxon Signed Ranks test. This was done for the overall scores for each efficacy variable.

In addition, subanalyses of Spearman's Rho correlation and 2-tailed analyses were performed between behavioral data obtained using the NPI, the FAB scores, and apathy scores.

Results are presented as mean changes from baseline with standard deviations, and *p* values are presented where appropriate.

## 3. Results

Of the 50 patients enrolled, 2 abandoned the study, so 48 patients have been fully studied and followed for 12 months (28 males, 20 women). They have been diagnosed as PDD in accordance with the complete fulfillment of the eight items of the diagnostic rating sheet for probable PDD, as recommended by the Movement Disorder Task Force [46], and their mean scores have been reported in Table 1. Their mean age is 70.4 ± 2.34 years old; their mean educational level is 8.5 ± 2.5 years of school attendance. The mean L-dopa equivalent dosage was 660 ± 130.5 mg/day. 42 patients received dopamine-agonists, 17 entacapone during their cure. Patients have been followed for a mean period of three years (2.5 ± 0.6 years) for motor disturbances, and they have been diagnosed as PDD during the first visit in our Unit. They have never assumed anticholinesterase inhibitors before our diagnosis. Patients were titrated in three months to receive the mean patch dose of transdermal Rivastigmine of 9.5 mg/24 hrs. The most salient side effects at every titration are nausea and disequilibrium; nobody refused the therapy and abandoned the study for conspicuous side effects. Heart frequency and blood pressure are measured at each visit but the caregivers have been instructed to measure by themselves at home three times a week.

Table 2 reproduced the summary of the most salient cognitive aspects, compared with a Wilcoxon Signed Rank test (12 months versus baseline).

Tables 3, 4, and 5 represent the behavior aspect resumed by NPI subscores, in order of their prevalence in the studied population, their relevance (the product of severity for frequency), and the derived caregiver stress parameters (as derived by the scores they gave).

Table 6 reflects a sum-up of the possible coexistence of more than 1 NPS for each patient, during the 12-month follow-up.

Table 7 reflects the qualitative results of apathy evaluation, as self-reported (AES-S) and clinically evaluated (AES-C). We have considered as apathetic a patient with a score of >37; all our patients satisfied the criteria, and we report the differences, according to a Wilcoxon rank signed test from the cutoff.

There is a slight, but significant decrease in global cognitive functions and in frontal executive functions, as reported in Table 2. From the very beginning, all the patients showed behavior symptoms, as reported in Table 3; the first evaluation indicates that 77% of patients manifested apathy, with a severe impact on daily living (8, as the product of frequency × severity, maximum score 12), and with a severe relevance for caregivers (4, in a scale from 0 to 5); the second more relevant symptom is anxiety (54% of patients), with an important impact in daily living (6, as the product of frequency × severity), and with a limited relevance for caregivers (2, in a scale from 0 to 5); the third symptom is depression (46% of patients), with a discrete impact in daily living (4, as the product of frequency × severity), and with a limited relevance for caregivers (2, in a scale from 0 to 5); the fourth symptom is hallucinations (42% of patients), with a limited impact on daily living (2, as the product of frequency × severity) and with a limited relevance for caregivers (1, in a scale from 0 to 5). It should be noted that all the other NPS symptoms have been reported, within a limited number of patients, and with limited consequences in daily living. Qualitative assessment of apathy (AES-S and AES-C) is online with this report, as shown in Table 7; as shown in Table 6, 25% of patients showed only one NPS symptom; 42% showed two or more NPS symptoms and 33% three or more. The mean total NPI composite score at baseline was 39.4 ± 12.1 and total caregiver distress score was 24.6 ± 11.1.

At 6-month evaluation, all the patients presented behavior symptoms, as reported in Table 4; the evaluation indicates that 62% of patients manifested apathy (decrease within group, 6 month versus baseline, with a Wilcoxon rank signed test, −7 ± 0.5, *p* < 0.001), with a severe impact on daily living (8, as the product of frequency × severity, maximum score 12), and with a severe relevance for caregivers (4, in a scale from 0 to 5, not significant from baseline); the second more relevant symptom remains anxiety (42% of patients; decrease within group, 6 months versus baseline, with a Wilcoxon rank signed test: −6 ± 0.8, *p* < 0.001), with a decreased impact on daily living (4, as the product of frequency × severity), and with a limited relevance for caregivers (1, in a scale from 0 to 5, *p* < 0.05 versus baseline); the third symptom is depression (35% of patients; decrease within group, 6 months versus baseline, with a Wilcoxon rank signed test: −4 ± 0.9, *p* < 0.001), with a modest impact on daily living (3, as the product of frequency × severity), and with a limited relevance for caregivers (1, in a scale from 0 to 5, *p* < 0.05); the fourth symptom is hallucinations (33% of patients; decrease within group, 6 months versus baseline, with a Wilcoxon rank signed test: −4 ± 0.9, *p* < 0.001), with a limited impact on daily living (2, as the product of frequency × severity), and with a limited relevance for caregivers (1, in a scale from 0 to 5, *p* < 0.05). It should be noted that all the other NPS symptoms have been reported, and caregivers reflect a relief of their distress; nonsignificant results have been found for agitation/aggression, euphoria, and aberrant motor behavior parameters, but they had very limited consequences in daily living and were minor cause of caregiver' distress. Moreover, as shown in Table 6, 21% of patients showed only one NPS symptom; 54% showed two or more NPS symptoms and 25% three or more. The mean total NPI composite score at baseline was 33.1 ± 17.1 (according to a Wilcoxon Signed rank test, within group, −6.3 ± 5.0, *p* < 0.05) and total caregiver distress score was 16.7 ± 7.1 (according to a Wilcoxon Signed rank test, within group, −5.9 ± 4.0, *p* < 0.05).

At 12-month evaluation, all the patients presented behavior symptoms, as reported in Table 5; results here have been compared within groups (12 months versus baseline and 6 months versus baseline); there is a general stability of the results; there is a slight decrease in the irritability scores, associated with a decrease in the disinhibition scores and in the euphoria scores (*p* < 0.001), in comparison with the results obtained at 6-month evaluation; apathy increased, up to 69% of patients (decrease within group, 12 month versus baseline, with a Wilcoxon rank signed test (−4 ± 0.7, *p* < 0.05)), with a severe impact on daily living (8, as the product of frequency × severity, maximum score 12) and with a severe relevance for caregivers (4, in a scale from 0 to 5, not significant from baseline and from 6 months); the second more relevant symptom remains anxiety, increasing up to 44% of patients up to 6 months (decrease within group, 12 months versus baseline, with a Wilcoxon rank signed test (−5 ± 0.8, *p* < 0.05)), with a stable impact on daily living (4, as the product of frequency × severity), but with a limited relevance for caregivers (1, in a scale from 0 to 5, *p* < 0.05 versus baseline, stable versus 6 month); the third symptom is depression, rising up to 40% from 6-month evaluation (decrease within group, 12 months versus baseline, with a Wilcoxon rank signed test (−3 ± 0.3, *p* < 0.05)), with a modest, stable, impact on daily living (3, as the product of frequency × severity), and with a limited relevance for caregivers (1, in a scale from 0 to 5, *p* < 0.05 versus baseline, stable versus 6 months); the fourth stable symptom is hallucinations (31% of patients, decrease within group, 12 months versus baseline, with a Wilcoxon rank signed test (−5 ± 0.4, *p* < 0.001)), with a limited impact on daily living (2, as the product of frequency × severity), and with a limited relevance for caregivers (1, in a scale from 0 to 5, not significant versus baseline and versus 6-month evaluation). Moreover, as shown in Table 6, 25% of patients showed only one NPS symptom; 58% showed two or more NPS symptoms and 17% three or more. The mean total NPI composite score at baseline was 28.7 ± 11.3 (according to a Wilcoxon Signed rank test, within group, versus baseline −4.4 ± 5.8, *p* < 0.05) and an increased total caregiver distress score, which was 20.3 ± 6.2 (according to a Wilcoxon Signed rank test, within group, +3.9 ± 1.1, *p* < 0.05).* Qualitative assessment of apathy (AES-S and C) is online with this report, as shown in Table 7 with a significant increase of AES-C, above the cutoff scores (according to a Wilcoxon Signed rank test, within group, versus baseline *+6.2 ± 2.1, *p* < 0.05*).*

Spearman's rank correlation analyses (made at 12 months) indicated that there was a significant correlation, in both the groups, between

1. NPI high scores and caregiver's distress (*r* = 0.78, *p* < 0.01);
2. NPI apathy score and AES-S and AES-C (*r* = 0.71, *p* < 0.01 and *r* = 0.78, *p* < 0.01, respectively);
3. FAB scores and apathy score (NPI and AES-C) (*r* = 0.75, *p* < 0.01 for NPI subscore and *r* = 0.79, *p* < 0.01, for AES-C).

## 4. Discussion

This work shares many points with the most recent published on the topic [5, 6, 42]; the principal and most significant of them is that NPS are the most salient aspects of PDD intellective disruption and the most relevant for their caregivers.

On the contrary of what merged from the other studies, where depression and anxiety are the most cited NPS, in this study we have found that apathy is the most constant NPS; its impact for frequency and severity is heavy and constant in the time and is onerous for the caregivers.

Rivastigmine works well and improves NPS symptoms, reducing caregivers' stress, in line with many other studies [5, 6, 54], in PD psychosis [55], in AD [56, 57], and in sVAD [20, 21] at the very beginning, and, in line with what has been previously described, we assist a first-step (6-month evaluation) global general amelioration of signs and symptoms in NPS, with a relief of caregiver's distress. That concerns practically all the NPS, a part from agitation and aberrant motor behavior; the results are so good to reflect on a significant decrease of NPI total score and an effective decrease on caregiver's burden. But even at this point, apathy, in our cases, maintains its hard impact, and for its frequency and severity it is related to caregiver's stress. Rivastigmine does not ameliorate that score. At 12-month evaluation, results in our study differ, even more, from the previous reported studies: there is a slight reduction of NPS signs, as demonstrated by the comparison with baseline results, but results are quite superimposable to what has been revealed at 6-month evaluation. On the contrary, caregivers burden increase significantly. That relates quite well with the qualitative perceive of apathy, as demonstrated by AES-S and AES-C, which remains stable from baseline throughout the entire follow-up. So, we can conclude that Rivastigmine might help for NPS symptoms in PDD, but its efficacy is limited in time, varies for symptoms, and does not have benefit for apathy in daily living.

Apathy increments in PDD the burden of the other motor and intellectual dysfunctions and worsens all the other behavioral symptoms. As reported in many dedicated studies, apathy is tightly related to PD or, better say, to a sufferance of the nigrostriatal pathway [13, 29, 58, 59]. Functional connectivity within the striatum and between striatal and ventrolateral prefrontal regions has been demonstrated in support of those studies, in patients with PD with high apathy compared to low [31], but results are not univocal, as they have been rejected by other studies [32, 33]. In general, many others are the brain networks related to apathy in PD, extending from the nigrostriatal up to frontal, cingulate, and limbic areas, precuneus, parietal inferior lobule, and so on (see data and literature in [22, 34]). If functional localization of networks underlying apathy is uncertain, even more complex is the neurochemical and pharmacological face. Hence, seeming quite conclusive that basal forebrain, striatum, parietal, and frontal cortex are strongly involved in apathy, three major neurotransmitters should be taken into account for its determination: acetylcholine, noradrenaline, and dopamine. An inverse correlation between catecholaminergic binding potential, indicative of a specific loss of dopamine and noradrenaline innervation, and apathy in PD was found in the bilateral ventral striatum [38]. But again, data are not so certain and are verified by studies in AD patients, although dopaminergic neurotransmission is thought to underline many goal-directed behaviors including addiction, and there is evidence for the efficacy of dopaminergic agents for apathy in AD [60]; there was no association between dopamine D2/D3 receptor density and apathy in AD [61]. We have no data, at the moment, for the studies of D2-D3 concentrations in PD or PDD with or without apathy.

Since many NPS respond to acetylcholinestease and butyrrylcholinesterase inhibitors, it can be documented a pivotal role of Ach in vivo. Rivastigmine works quite well, at least at the very beginning for NPS, but not for apathy. What seems highly probable is that in PDD there is a very precarious equilibrium between dopamine and Ach and that disequilibrium might potentiate the resistance of specific symptoms such as apathy, probably determined by the alteration of multineurotransmitters synaptic networks.

Our study has some strengths:

1. We enrolled first-diagnosed PDD patients who began Rivastigmine in our study and have been studied for 12 months.
2. All the patients can be fully examined and have a strenuous assistance of a caregiver, who is the other actor of the study.
3. We employed some similar method as those described by Oh et al. (2015-A) [6] and implement some other measures, but as Oh et al., [6] we strictly surveyed the pharmacological intake of our patients in order to avoid any other interference bias.
4. All the patients have been supplied by transdermal Rivastigmine patch.

However, our study has several weaknesses:

1. It is a single center study, and the number of patients is very small to infer definite results.
2. It is an open-label and not blinded study.
3. It has no pathological confirmation.

Data deriving from our study suggest that

1. apathy should be considered from the very initial phases of PD (and PDD) by devoted neuropsychological instrument;
2. multireceptor approach should be employed to treat it, so many potential sites could be employed, such as SSRI, NARI, dopaminergic agonists, and probably modulating also Ach;
3. apathy should be discussed with and explained to patients and to caregivers: it might help to confront it better;
4. neurologists should think more overtly about apathy, in order to understand it and possibly treat it; apathy inside a specific disease, such as PD, or AD, or sVAD might become something different. It must be said, forwardly, that, as well as in AD, it seems difficult to find a neurodegenerative complex clinical condition, such as PDD, in which apathy is an isolated symptom. AS in our study, even if devotedly constructed to define it, apathy can coexist with other NPS, and therefore, its anatomical and biochemical core could be modified and worsened, by the interference of many other neurochemical substrates, which underline opposite symptoms, such as anxiety, aggression, and euphoria.

## 5. Conclusion

In conclusion, our study confirmed some results of many other precedent studies, on the positive results of Rivastigmine for the reduction of NPS symptoms, but we are more circumspect on its effect on apathy, suggesting that polyvalent and multireceptor treatment should be desirable and employed; larger, placebo-controlled studies should be required to define, adequately combat, and give long-lasting relief to such a difficult symptom, as apathy is.

## Figures and Tables

**Table 1:** Cognitive parameters. Values are mean (SD). NS = not significant. ^*∗*^MMSE corrected for the adjustments according to age and education.

	Recruitment
MMSE^*∗*^	23.7 (0.6)
Pill Questionnaire	2.1 (0.2)
Attention, months reversed	3.1 (0.4)
Lexical fluency	8.2 (0.7)
MMSE pentagons	0.1 (0.1)
3-word recall	2.1 (0.3)
GDS-15	3.6 (0.5)

**Table 2:** Cognitive parameters at baseline and at 12 months. Values are mean (SD). NS = not significant. ^*∗*^MoCA are reported as raw scores, and in square brackets corrected for the adjustments according to age and education expressed as years of schooling-Conti et al., 2015; Santangelo et al., 2015.

	Baseline	12 months	Within groups (12 months versus baseline) *p* value
MoCA^*∗*^	24.1 (0.9) [22.01 (0.8)]	21.3 (0.2) [18.1 (0.7)]	*p* < 0.05
FAB total score	8.2 (0.5)	7.1 (1.3)	*p* < 0.05
Analogies	1.1 (0.2)	1.1 (0.6)	*p* < 0.05
Phonemic fluency	1.2 (0.2)	0.9 (0.5)	NS
Motor series	2.1 (0.7)	1.6 (0.2)	*p* < 0.05
Contrast instructions	2.1 (0.8)	1.4 (0.5)	*p* < 0.05
Go/no-go	0.6 (0.5)	0.2 (0.4)	*p* < 0.05
Prehension behavior	1.1 (0.9)	0.9 (0.8)	NS

**Table 3:** Baseline NPI results.

Subitems NPI	Number of patients/48 (%)	Frequency × severity	Caregiver distress
hallucinations	20 (42%)	2	1
Delusions	10 (21%)	1	1
Agitation/aggression	5 (10%)	2	1
Dysphoria/depression	22 (46%)	4	2
Anxiety	26 (54%)	6	2
Irritability	7 (15%)	4	2
Disinhibition	2 (4%)	2	2
Euphoria	3 (6%)	2	2
Apathy	37 (77%)	8	4
Aberrant motor behavior	3 (6%)	2	2
Sleep behavior change	6 (12%)	4	3
Appetite change	2 (4%)	2	2

**Table 4:** 6-month NPI results; in the first and third rows it has been reported the within group comparison with baseline.

Subitems NPI	Number of patients/48 (%) within groups (6 months versus baseline)	Frequency × severity	Caregiver distress
hallucinations	16 (33%) (*p* < 0.001)	2	1 (NS)
Delusions	7 (15%) (*p* < 0.05)	1	1 (NS)
Agitation/aggression	4 (8%) (NS)	2	1 (NS)
Dysphoria/depression	17 (35%) (*p* < 0.001)	3	1 (*p* < 0.05)
Anxiety	20 (42%) (*p* < 0.001)	4	1 (*p* < 0.05)
Irritability	4 (8%) (*p* < 0.001)	3	1 (*p* < 0.05)
Disinhibition	1 (2%) (*p* < 0.001)	2	1 (*p* < 0.05)
Euphoria	2 (4%) (NS)	2	1 (*p* < 0.05)
Apathy	30 (62%) (*p* < 0.001)	8	4 (NS)
Aberrant motor behavior	2 (4%) (NS)	2	2 (*p* < 0.05)
Sleep behavior change	4 (8%) (*p* < 0.001)	2	3 (*p* < 0.05)
Appetite change	1 (2%) (*p* < 0.001)	2	2 (*p* < 0.05)

**Table 5:** 12-month NPI results. In the first and third rows it has been reported the within group comparison with baseline and with 6-month results.

Subitems NPI	Number of patients/48 (%) within groups (12 months versus baseline) (12 months versus 6 months)	Frequency × severity	Caregiver distress within groups (12 months versus baseline) (12 months versus 6 months)
Hallucinations	15 (31%) (*p* < 0.001) (NS)	2	1 (NS) (NS)

Delusions	8 (17%) (*p* < 0.05) (NS)	1	1 (NS) (NS)

Agitation/aggression	3 (8%) (NS) (NS)	2	1 (NS) (NS)

Dysphoria/depression	19 (40%) (*p* < 0.05) (NS)	3	1 (*p* < 0.05) (NS)

Anxiety	21 (44%) (*p* < 0.001) (NS)	4	1 (*p* < 0.05) (NS)

Irritability	1 (2%) (*p* < 0.001) (*p* < 0.001)	3	1 (*p* < 0.05) (NS)

Disinhibition	0 (0%) (*p* < 0.001) (*p* < 0.001)	0	1 (*p* < 0.05) (NS)

Euphoria	0 (0%) (*p* < 0.001) (*p* < 0.001)	0	1 (*p* < 0.05) (NS)

Apathy	33 (69%) (*p* < 0.05) (NS)	8	8 (NS) (NS)

Aberrant motor behavior	1 (4%) (NS) (NS)	1	1 (*p* < 0.05) (NS)

Sleep behavior change	5 (10%) (*p* < 0.001) (NS)	3	2 (*p* < 0.05) (NS)

Appetite change	1 (2%) (*p* < 0.001) (NS)	1	1 (*p* < 0.05) (NS)

**Table 6:** A synopsis of the presence of a single or more than one NPS for each patient, during follow-up.

	3 or more NPS num pts/48 (%)	2 or more NPS num pts/48 (%)	1 NPS num pts/48 (%)
NPI baseline	16 (33%)	20 (42%)	12 (25%)
NPI 6 months	12 (25%)	26 (54%)	10 (21%)
NPI 12 months	8 (17%)	28 (58%)	12 (25%)

**Table 7:** Apathy scores, AES-C, clinician rated apathy evaluation scale; AES-S, self-report rated apathy evaluation scale. Values are mean (SD). NS = not significant. We report the differences from the cutoff score.

	Baseline cutoff > 37	12- month cutoff > 37	Within group baseline/12 months
AES-S	+16.3 (4.1)	+ 19.9 (2.1)	NS
AES-C	+15.5 (3.7)	+21.5 (2.7)	*p* < 0.05
